# Assessing the predictability of existing water-to-enamel geolocation models against known human teeth

**DOI:** 10.1038/s41598-021-95153-w

**Published:** 2021-08-02

**Authors:** Momoko Ueda, Lynne S. Bell

**Affiliations:** School of Criminology, Centre for Forensic Research, Simon Fraser University, Burnaby, BC Canada V5A 1S6

**Keywords:** Stable isotope analysis, Archaeology, Biological anthropology, Geochemistry

## Abstract

Stable isotope analysis of human tissues has become a valuable tool for mapping human geolocation. This study adds to the existing knowledge of the relationship between oxygen stable isotopes in human enamel and drinking water by presenting enamel oxygen values in clinic-extracted human dental enamel with known provenance. The results from this study indicate that the theoretical isotopic relationship between enamel and drinking water oxygen is weak at the city and country-level. Differences of up to 15‰ were observed between predicted drinking water oxygen values using existing models and observed values, highlighting the complexity of using water/enamel conversion equations. The lower isotopic boundary of enamel oxygen values is now understood for Metro Vancouver at *δ*^18^O_c(VPDB)_ = – 11.0‰ and presents the possibility of using stable isotope analysis as an exclusionary tool where individuals falling below threshold value can be identified as non-local. Overall, this study’s results support the development of geographical reference maps for human enamel oxygen.

## Introduction

Stable oxygen isotope analysis is increasingly becoming an essential tool for localizing region-of-origin of unknown human remains in both archaeological and forensic settings^1–3^. The use of oxygen stable isotope analysis is premised on the foundational understanding that human tissues reflect stable isotope compositions of the environment in which they resided during tissue formation. Human enamel is one of the more commonly analyzed tissues as it is resistant to degeneration because of its highly mineralized tissue composition (approximately 95%) along with the organic (1%) and water (4%) contents^4,5^. The linear relationship between skeletal bioapatite and drinking water was initially investigated in the 1980s to understand paleoclimate^6–8^. Although not initially studied for this purpose, the water/bioapatite conversion equations became the basis for utilizing oxygen isotope analysis to reconstruct human mobility patterns. Oxygen isotope compositions of human skeletal tissues reflect the oxygen isotope compositions of drinking water, which is influenced by local precipitation water where oxygen isotope compositions vary geographically^9,10^. Several isotope distribution maps, otherwise known as isoscapes, exist for precipitation and drinking water oxygen, and are developed using spatial analytical methods to predict the isotopic variation across a geographical area^11–13^. The water/enamel conversion equations are then applied to derive human tissue oxygen isoscapes. Human enamel oxygen isoscapes developed through this approach exists for the United States^14^ and the Circum-Caribbean region^15^. Oxygen isotope compositions of enamel from unknown human remains can thus be analyzed to place individuals on a geographical map. Individuals are then identified as having a local or non-local origin, depending on how well the estimated drinking water values agree or disagree with values from the targeted geographical area. There are, however, noticeable differences in slopes and intercepts of water/enamel conversion equations^6–8,14,16–23^. Such differences in the proposed equations have real-world implications where calculated drinking water values from enamel oxygen values can result in offsets^16,21,24–26^. Accurate reconstruction of an individual’s life history is important for reaching potential identifications of unknown human remains in both human archaeological material and forensic casework. The application to forensics is particularly important as it helps connect missing person files to unidentified remains. Without good validation studies in this area, the current predictive power of isotopic geolocation is questionable.

When considering obligate drinkers such as humans, the linear relationship between water and enamel oxygen can be explained by the direct relationship between drinking water oxygen and blood water oxygen^6–8^. Human enamel bioapatite, the inorganic component of the human tooth, precipitates in isotopic equilibrium with body water in and around a constant temperature of 37 °C. Whilst the primary source of oxygen input to the body water itself is imbibed water, food water, and inhaled atmospheric oxygen, body water mainly reflects the isotopic composition of oxygen in drinking water^27^. This is because atmospheric oxygen has a constant isotopic composition and does not lead to isotopic variability of body water, although making up the largest contribution of body water oxygen^7^. Further, food water oxygen contributes little to body water^16^. Oxygen isotope compositions of drinking water are shown to follow the pattern of local precipitation, which differs geographically due to local physiographic factors such as latitude, altitude, and the distance from the coast^9,10^. There is a general latitudinal pattern of stable oxygen isotope values decreasing with increasing latitude^28^. Studies have confirmed this linear relationship between enamel and water oxygen by analyzing oxygen isotopes from both the phosphate and carbonate components of the enamel bioapatite^6–8,14,16,17,19–24^.

**Figure 1:**
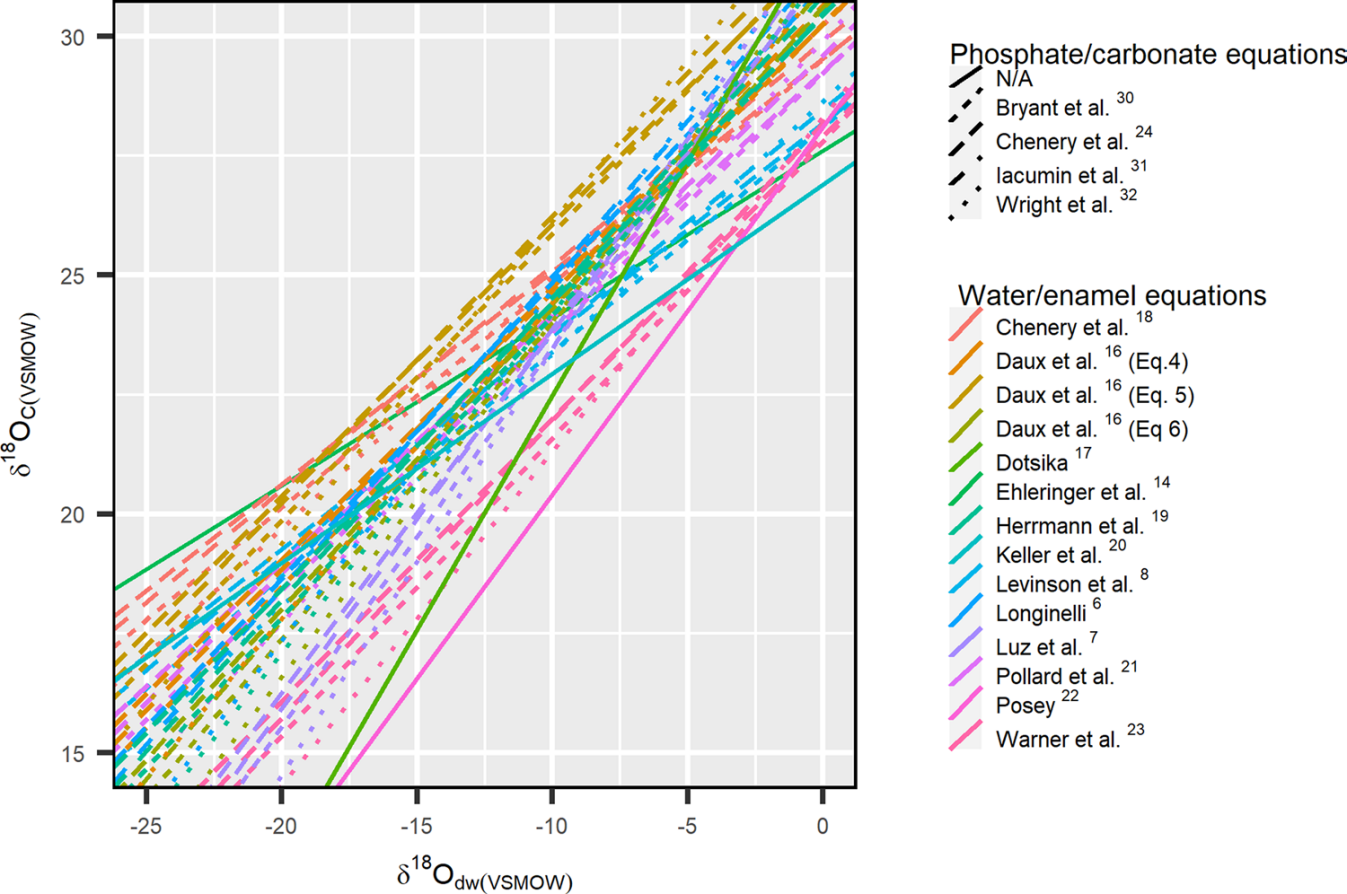
Existing water/enamel conversion equations are shown as the relationship between drinking water oxygen values (*δ*^18^O_dw_) and enamel carbonate oxygen values (*δ*^18^O_C(VSMOW)_). This graph aims to provide a visual representation of the various water/enamel conversion equations that exist to date and to show the general spread of slopes and intercepts, rather than for interpretation. Phosphate/carbonate conversion equations were applied to water/enamel equations based on enamel phosphate oxygen. Solid lines indicate the equations that allowed for direct conversions between *δ*^18^O_dw_ and *δ*^18^O_C(VSMOW)_ values without requiring the use of an additional phosphate/carbonate conversion. The linetypes indicate the use of different phosphate/carbonate conversion equations.

Enamel bioapatite contains carbonate anions substituted at the phosphate sites, allowing for oxygen isotope analysis to be conducted on either phosphate or carbonate oxygen^29^. The isotopic spacing between oxygen isotopes in phosphate and carbonate is well understood and makes conversions between phosphate oxygen values and carbonate oxygen values possible^24,30–32^. Although the phosphate/carbonate equations have slightly differing slopes and intercepts, the uncertainties associated with the use of phosphate/carbonate conversion equations have been shown to fall within the errors of oxygen analysis itself^24^. Thus, the differences are not of concern here. The existing water/enamel conversion equations are shown in Fig. 1. More recent studies have reported oxygen values analyzed from the carbonate component of bioapatite^14,17,20,22,23^, and thus equations based on phosphate oxygen have been converted to carbonate oxygen values through the use of existing phosphate/carbonate oxygen conversion equations. Direct comparisons of equations should be proceeded with caution due to uncertainties in analyzed oxygen isotope values resulting from differences in analytical methodologies between laboratories^33^; however, it is evident that slope and intercepts of the existing water/enamel equations differ greatly (Fig. 1).

The general methodological approach behind each of the existing water/enamel conversion equations is summarized in Table 1. Besides Longinelli’s^6^ equation based on historical samples, more recent equations have been based on modern individuals^8,14,17,19,20,22,23^. The collection of modern samples with known provenance is vital for allowing the appropriate matching of enamel values with drinking water values from the same geographical area. However, an important difference amongst the existing studies is in defining the term “drinking water.” Conversion equations were based on various drinking water samples, including meteoric water^6,19^, estimated precipitation water^7,16^, river water^20^, spring water^17^, or tap water^14,16,22,23^. Given the understanding that the oxygen isotope composition of precipitation is reflected in human drinking water oxygen, it seems reasonable to rely on precipitation data that is readily available through the Global Network of Isotopes in Precipitation, a monitoring network with collection stations located worldwide^34^, or through the Online Isotopes in Precipitation Calculator (OIPC)^35^. The OIPC can be utilized to estimate precipitation oxygen isotope values for any geographical location by feeding latitudinal, longitudinal, and elevational data. However, an important consideration that must be made is that the degree to which precipitation oxygen values translate to human drinking waters in modern society is dependent on several factors such as the type of drinking water sources, climatic characteristics, and water infrastructure^36,37^. There is a heightened reliance on complex water distribution systems for safe drinking water delivery and is becoming essential for high density and expanding metropolitan regions^38,39^. Tap water distribution systems often draw water from multiple water sources or geographically distant areas with varying physiography^40^ to meet growing communities’ demands. This ultimately leads to significant offsets in tap water oxygen values compared to that of local precipitation values. Thus, it is essential to analyze water samples that accurately represent the isotopic composition of oxygen in consumed drinking water for pairing with enamel oxygen. This is vital as water/enamel conversion equations are importantly utilized as a means to localize unknown human remains.

**Table 1:** Summary of studies on predictive models.

Study	Time period	Sampled material	Geographical scale	Definition of drinking water	Oxygen analysis
Longinelli^6^	End of nineteenth century	Human bone	Global	Meteoric water	Phosphate (BiPO_4_)
Luz et al.^7^	Unspecified	Human tooth	Global	Estimated precipitation and tap water	Phosphate (BiPO_4_)
Levinson et al.^8^	Modern	Human tooth	Global	Unspecified	Phosphate (BiPO_4_)
Daux et al.^16^ (Eq. 4)	Modern and historical (Greenland)	Human 2nd and 3rd molars	Global	Tap water (OPIC for Disko Bay)	Phosphate (Ag_3_PO_4_)
Daux et al.^16^ (Eq. 5)	Modern and historical (Greenland)	Human 2nd and 3rd molars	Global	OIPC precipitation estimate	Phosphate (Ag_3_PO_4_)
Ehleringer et al.^14^	Modern	Human tooth	Country (US)	Tap water	Carbonate
Posey^22^	Modern	Human tooth (incisors, canines, premolars, molars)	Region (Middle East)	Tap water	Carbonate
Herrmann et al.^19^	Modern	Human tooth	Country (US)	Meteoric water	Phosphate (Ag_3_PO_4_)
Keller et al.^20^	Modern	Human 3rd molars	Country (US)	River water	Carbonate
Warner et al.^23^	Modern	Human 3rd molars	State (Mississippi, US)	Tap water	Carbonate
Dotsika^17^	Modern	Human 2nd molars	Country (Greece)	Spring water	Carbonate

There is an increased understanding of the spatial variations in stable isotope values of tap water across large geographical areas such as the conterminous United States^41,42^, Mexico^43^, France^44^, South Africa^13^, Korea^45^, and China^46^. However, very little is known for defined metropolitan areas and whether a single tap water oxygen value represents an entire city, especially those relying on multiple water sources. A study of the distribution of hydrogen and oxygen isotope values in tap water across the entire regional district of Metro Vancouver showed that tap water values uniquely followed the tap water distribution system of Metro Vancouver^47^. The study showed the importance of tap water distribution systems on the spread of hydrogen and oxygen isotope values of tap water across a single geographical locale, in which the isotope values closely followed the pattern of the water distribution pipelines. Most Metro Vancouver municipalities rely on the three major watersheds located north of the region, but a smaller proportion of the residents consume tap water from groundwater sources, which was isotopically distinct from the other water sources. Thus, the study indicated the importance of understanding tap water distribution systems for the defined geographical area to ensure that collected tap water samples cover every water source that contributes to the overall tap water system, especially if those sources are isotopically distinct. Another high resolution tap water study for Salt Lake Valley, Utah showed similar results where spatiotemporal variation was noted in tap water isotope values across the region^48^.

An alternative approach to the use of conversion equations has been proposed where Pellegrini et al.^26^ created a tissue-based isoscape from enamel phosphate oxygen for Britain based on a large sample of archaeological remains. This approach eliminates the need for water-enamel conversion equations as the tissue isotopes themselves were used for mapping. However, the challenge with such an approach is that it necessitates the extensive sampling of human enamel and therefore requires a significant amount of time for sample collection. And perhaps more importantly, the life-history or provenance of those individuals is unknown during the period of enamel formation. The current approach to the development of tissue isoscapes does not require an extensive collection of enamel samples since it is based on drinking water isoscapes paired with modern samples with known provenance. Again, this approach requires the use of conversion equations with various proposed slopes and intercepts, without a clear understanding as to which equation best represents the water/enamel relationship.

The purpose of this study is to validate and refine the water/enamel conversion model by carefully matching oxygen isotope values in recently extracted enamel with known tap water values from cities across Metro Vancouver. Tap water samples were collected directly from Metro Vancouver tap water sources every month over a 12 month period. It is important to emphasize that water samples were strategically sampled to capture the entire spread of oxygen isotope compositions of drinking water for Metro Vancouver by referring to the detailed isotope study of tap water distribution^47^. Biographically known enamel oxygen results from this study will also be compared against existing water/enamel conversion models to determine how well the existing models perform against known modern enamel and drinking water samples.

## Methods

### Ethics permission

This research project has been approved by the Associate Director, Simon Fraser University Office of Research Ethics, on behalf of the Research Ethics Board under the study number [2015s0125]. Informed consent was obtained from all subjects, and all experiments were performed in accordance with University Policy R20.01 (http://www.sfu.ca/policies/gazette/research/r20-01.html).

### Collection and analysis of tooth samples

154 empty vials were sent out to dental clinics across Metro Vancouver during the years 2015 to 2017. 126 vials were returned with extracted human molar samples comprised of first, second, and third molars. Molars were placed in 20 mL scintillation vials labelled with sample identifiers following the extraction and returned to the laboratory for storage. Immense care was taken to obtain information from individuals on location of residence and any relocations from birth to 25 years which covers the entire duration of enamel formation (Table 2). This information was vital to ensure accurate matching of enamel oxygen to drinking water oxygen.

**Table 2:** Summary of molar formation^82^.

	Tooth	Formation of tissue	Crown completion	Root completion
Permanent maxillary teeth	First	Birth	2.5–3 years	9–10 years
	Second	2.5–3 years	7–8 years	14–16 years
	Third	7–9 years	12–16 years	18–25 years
Permanent mandibular teeth	First	Birth	2.5–3 years	9–10 years
	Second	2.5–3 years	7–8 years	14–15 years
	Third	8–10 years	12–16 years	18–25 years

Collected molars were cleansed with tap water and dried for at least 24 h before drilling. Core enamel was extracted into powdered form (~ 1 mg) by gentle abrasion with a diamond-tipped hand-held drill (Dremel). The enamel’s external surface was mechanically removed and discarded prior to sampling core enamel to ensure that sampled areas were devoid of dental calculus and overt caries. Care was taken to ensure no dentine was sampled, and the drill was cleaned of any enamel powder with compressed air between each sample. Enamel powder was prepared after Lee-Thorp et al.^49^. The powder was soaked in 50% sodium hypochlorite for 45 to 60 min, centrifuged, and rinsed with distilled water. The powder was then reacted with 0.1 M acetic acid for 5 to 15 min, thoroughly rinsed with distilled water then freeze-dried.

Samples were inserted into 12 ml borosilicate glass vials with a septum-contained screw top lid (Labco, Ceredigion UK) and place in a temperature-controlled (72 °C) sampler tray. To remove atmospheric air the tubes were flushed with helium by using the CTC Analytics A200S autosampler (Switzerland). Depending on the sample size, five to seven drops of 72 °C acid (85% orthophosphoric acid and phosphorus pentoxide, specific gravity of solution = 1.92) were added to each vial manually through the septum with a 1 ml syringe. Samples were analyzed after being left to react for three hours^49,50^. The autosampler was used to sample the gas that evolved from each reaction and subsequently passed to the Themo Finnigan model II GasBench (Germany). The sample gas was then went through the Nafion water removal unit. To separate the gas compounds, the sample gas passed through the “Poraplot Q” GC column followed by another Nafion water trap. The gas then passed through the GasBench to a Delta Plus XP isotope ratio mass spectrometer (Thermo electron, Bremen, Germany), which is computer-controlled by Isodat software. The gas flow gave 8 sample peaks and 5 reference peaks. The reference gas (CO_2_) with 99.995% purity was introduced into the mass spectrometer through the Isodat software-controlled GasBench^50^.

Oxygen analyses were carried out on the structural carbonate of tooth enamel. Isotopic results are reported in conventional *δ*—notation in units of per mil (‰) with reference to Vienna Pee Dee Belemnite (VPDB) carbonate and calculated according to the equation:

$${\delta }^{18}{O}_{std} = \frac{R{({}{}^{18}O/{}{}^{16}O)}_{P}}{R{({}{}^{18}O/{}{}^{16}O)}_{std}} - 1$$

where R = ratio of the abundance of heavier ^18^O to lighter ^16^O isotopes in substance P and std = standards^51^. The isotopic compositions of enamel carbonate oxygen were measured in four runs. Each run included 21 to 39 samples and four replicates each of two international reference materials (NBS 18 and NBS 19) and an internal standard (Cavendish Marble). Measured oxygen (*δ*^18^O) values were normalized through an offset correction using the International Atomic Energy Agency (IAEA) accepted *δ*^18^O values of for NBS 18 and NBS 19 at – 23.03 and – 2.2‰, respectively, and a long-term accepted value of – 8.95‰ for Cavendish Marble. Analytical precision was ± 0.2‰ as calculated by taking the standard deviations for the three standards (NBS 18, NBS 19 and Cavendish Marble) for each run (standard deviation of repeated measurements (n = 16) of the internal standard of Cavendish Marble was 0.2‰).

### Collection and analysis of annualized Metro Vancouver tap water oxygen values

Two treated tap water samples were collected monthly at the two main water treatment plants in Metro Vancouver (MV), the Seymour-Capilano Filtration Plant (SCFP) and the Coquitlam Water Treatment Plant (CWTP), from June 2017 to May 2018. Samples were collected in air-tight 8-dram borosilicate glass vials and stored in a refrigerator to prevent evaporation. 3 of the 48 collected tap water samples were discarded due to leakage during transport, leaving only single measurements for June 2017 and May 2018 at CWTP and for October 2017 at SCFP. A total of 45 tap water samples were thus available for analysis. The water samples were analyzed for oxygen through the Los Gatos Triple Liquid Water Isotope Analyzer (TLWIA-45-EP 2013) connected to CTC Analytics LEAP Technology PAL liquid auto-sampler and checked against Los Gatos Standards 1A, 2A, 3A, 4A and 5A. Instrumental precision was ± 0.1‰.

MV tap water is delivered to municipalities through interconnected water pipes originating from the three main MV watersheds. Several municipalities additionally draw tap water from wells^52^. Although the water distribution system is well monitored, the source of tap water delivered to the municipalities through the interconnected water pipes can vary even by the hour. As a result, the exact fractional contributions of end-member water sources to the overall municipal tap water supply are unknown. The fractional contribution (*f*) of each water source (*a, b, c, d*) to the total water supply can be expressed as:

$$1 = \, f_{(a)} + f_{(b)} + f_{(c)} + f_{(d)}$$

Source information of tap water samples collected and reported in Ueda and Bell’s^47^ study was used to determine each water source’s potential fractional contribution to MV municipality water supply. The fractional contributions of each water source were then utilized to calculate the mean tap water oxygen (*δ*^18^O_tap_) values for each MV municipality:

$$\delta^{18} O_{tap} = \delta^{18} O_{(a)} * \, f_{(a)} + \delta^{18} O_{(b)} *f_{(b)} + \delta^{18} O_{(c)} * \, f_{(c)} + \delta^{18} O_{(d)} * \, f_{(d)}$$

Drinking water from Capilano and Seymour watersheds was treated under two separate facilities until the Seymour–Capilano Twin Tunnel construction in 2015, which now connects water from the Capilano watershed to the Seymour-Capilano Filtration Plan before distribution to the municipalities^52^.

### Data collection of drinking water oxygen values for individuals placed outside of Metro Vancouver

Isotopic data on drinking water oxygen (*δ*^18^O_dw_) values for cities outside of MV were retrieved from existing publications for comparisons with enamel carbonate oxygen (*δ*^18^O_c_) values. The preferred drinking water type was tap water with known source water information. Tap water data without any indications of the actual source of tap water were also collected but with the understanding that it may represent mixed water sources if supplied by multiple water sources. The temporal representation of the data was also noted, whether annual or a one-time collection. Isotopic data of actual or possible source water were taken as an alternative when tap water data did not exist. Average values were calculated if more than one *δ*^18^O_tap_ value was reported for a given city. Information on tap water sources was collected through municipal or governmental websites and from published studies. The OIPC^35^ and the Regionalized Cluster-based Water Isotope Prediction (RCWIP) database^11,34,53^ were utilized to estimate local *δ*^18^O_OIPC_ and *δ*^18^O_RCWIP_ values, respectively.

### Statistical analysis

The pairwise t-test was used to test for differences between estimated precipitation water oxygen values retrieved from the two databases at the α = 0.05 level. The ordinary least squares (OLS) method was utilized to measure the correlation between *δ*^18^O_c_ and *δ*^18^O_dw_ values with adjusted r-squared ($\overline{R }$^2^) as the goodness-of-fit measure^54^. Coplen’s^55^ equation was utilized for any conversions from VPDB to Vienna Standard Mean Ocean Water (VSMOW). One-way analysis of variance (ANOVA) tests were performed to compare means of *δ*^18^O_c_ between cities, water sources, and countries. When ANOVA assumptions were not met, the Kruskal–Wallis rank sum test^56^ was performed as a non-parametric alternative to one-way ANOVA. Shapiro–Wilk’s^57^ method was used to test for normality of data. All mapping and statistical analyses were conducted on R version 4.0.3^58^.

### Water/enamel modelling and assessment of predictability

*δ*^18^O_dw_ values were predicted from measured *δ*^18^O_c(VSMOW)_ values using existing water/enamel conversion equations. When direct *δ*^18^O_c_ to *δ*^18^O_dw_ conversions were not possible, *δ*^18^O_c_ values were first converted to enamel phosphate oxygen (*δ*^18^O_p_) values using one of the phosphate/carbonate conversion equations listed in Table 3. The mean squared deviation (MSD)^59,60^ between predicted and measured drinking water oxygen values were calculated to measure the predictability of existing water/enamel conversion equations. MSD was calculated as

**Table 3:** Existing conversion equations.

Conversion	Study	Equation	n	r^2^
VPDB/VSMOW	Coplen^55^	*δ*^18^O_x/VSMOW_ = 1.03091 * δ*^18^O_x/VPDB_ + 30.91		
		*δ*^18^O_x/VPDB_ = 0.97001 * δ*^18^O_x/VSMOW_ – 29.99		
*δ*^18^O_p_/*δ*^18^O_c_	Iacumin et al.^31^	*δ*^18^O_p(SMOW)_ = 0.98 * δ*^18^O_c(SMOW)_ – 8.5	31	0.98
	Bryant et al.^30^	*δ*^18^O_c_ (± 1.3) = 1.02 (± 0.04) * δ*^18^O_p_ + 8.3 (± 0.7)	42	0.99
	Wright et al.^32^	*δ*^18^O_p(SMOW)_ = 0.899 * δ*^18^O_c(PDB)_ + 21.397		
	Chenery et al.^24^ (cold, temperate and warm humid climates)	*δ*^18^O_p_ = 1.0322 (± 0.008) * δ*^18^O_c_ – 9.6849 (± 0.187)	51	0.98
	Chenery et al.^24^ (hot-arid climates)	*δ*^18^O_p_ = 1.122 * δ*^18^O_c_ – 13.73	38	0.91
*δ*^18^O_p_/*δ*^18^O_dw_	Longinelli^6^	*δ*^18^O_p_ = 0.64 * δ*^18^O_w_ + 22.37	10	0.98
	Luz et al.^7^	*δ*^18^O_p_ = 0.78 * δ*^18^O_dw_ + 22.7	6	0.97
	Levinson et al.^8^	*δ*^18^O_p_ = 0.46 * δ*^18^O_dw_ + 19.4	40	0.93
	Daux et al.^16^ (Eq. 4)	*δ*^18^O_dw_ = 1.73 (± 0.21) * δ*^18^O_p_ – 37.25 (± 3.55)	12	0.87
	Daux et al.^16^ (Eq. 5)	*δ*^18^O_dw_ = 1.70 (± 0.22) * δ*^18^O_p_ – 39.28 (± 3.68)	12	0.88
	Daux et al.^16^ (Eq. 6) *combined equation	*δ*^18^O_dw_ = 1.54 (± 0.09) * δ*^18^O_p_ – 33.72 (± 1.51)	42	0.87
	Chenery et al.^18^ *corrected Levinson et al.^8^	*δ*^18^O_p_ = 0.46 * δ*^18^O_dw_ + 20.8	40	
	Pollard et al.^21^ *superset	*δ*^18^O_p_ = 0.531 * δ*^18^O_dw_ + 20.52	94	0.907
	Herrmann et al.^19^	*δ*^18^O_p_ = 0.62 * δ*^18^O_dw_ + 21.74	45	0.63
	Warner et al.^23^	*δ*^18^O_dw_ = 1.64 * δ*^18^O_p_ – 31.35	27	0.54
*δ*^18^O_c_/*δ*^18^O_dw_	Ehleringer et al. ^14^	*δ*^18^O_c(VSMOW)_ = 0.350 * δ*^18^O_dw_ + 27.6	5	0.85
	Posey^22^	*δ*^18^O_c(VSMOW)_ = 0.77 * δ*^18^O_dw_ + 28.1	58	0.70
	Keller et al.^20^	*δ*^18^O_c(VSMOW)_ = 0.396 * δ*^18^O_dw_ + 26.9	156	0.81
	Dotsika^17^	*δ*^18^O_dw_ = 1.020 * δ*^18^O_c_ – 32.941	16	0.87

$${\text{MSD}}=\frac{1}{\mathrm{n}}{\sum }_{k=1}^{n}{{(x}_{k}-{y}_{k})}^{2}$$

where *x* = predicted and *y* = measured values. MSD measures the degree of deviation from the equality line, where slope = 1 and intercept = 0, and was preferred over the more commonly used statistic of mean squared error that measures the mean deviation from the regression line rather than the equality line. MSD can further be partitioned into three separate components and is the sum of squared bias (SB),

$${\text{SB }} = \, ( \overline{x}{-}\overline{y})^{{2}}$$

non-unity slope (NU) where *b* = $\sum {x}_{n}{y}_{n}/\sum {x}_{n}^{2}$,

$$NU = (1 - b)^{2} *(\sum x_{n}^{2} *1/n)$$

and lack of correlation (LC) where *r* = ($\sum {x}_{n}{y}_{n})$^2^/$\sum {x}_{n}^{2}\sum {y}_{n}^{2}$,

$$LC = (1 - r^{2} )*(\sum y_{n}^{2} *1/n)$$

The models or sets of models were then ranked according to MSD to determine the best performing models for predicting *δ*^18^O_tap_ or *δ*^18^O_OIPC_ values from observed *δ*^18^O_c_ values.

## Results

**Figure 2:**
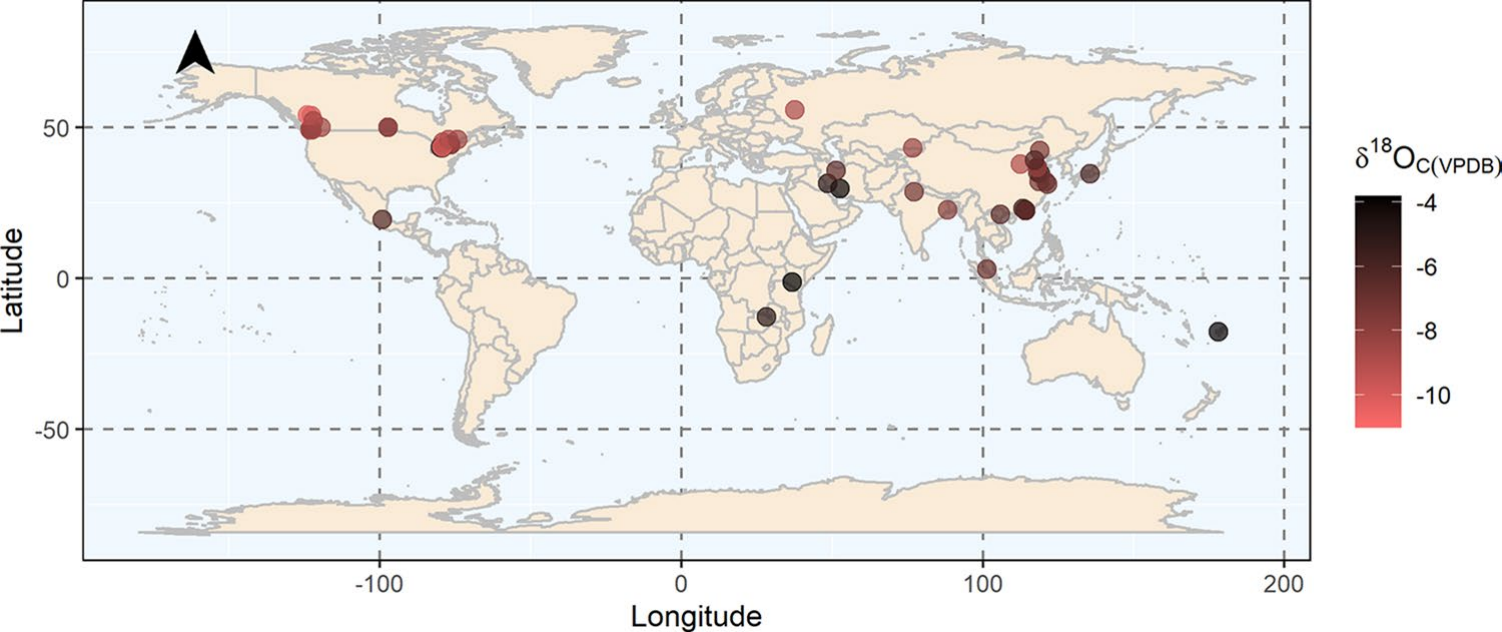
Locations of residence during tissue formation with the corresponding *δ*^18^O_C(VPDB)_ values for all collected samples. This map was generated using R version 4.0.3 (https://www.R-project.org/)^58^.

The collection of enamel samples from dental clinics across Metro Vancouver (MV) resulted in a globally representative dataset. Samples included individuals from 14 countries (Fig. 2), reflecting Metro Vancouver’s high fluidity in international migrations^61^. Of the collected tooth samples, enough enamel powder was generated from 103 samples for isotopic analysis. Two samples returned no oxygen isotopic signatures signifying the absence of hydroxyapatite, which could be attributed to the presence of dental porcelain visually akin to dental enamel. As it is important to obtain information related to the geographical location in which the individual lived during enamel mineralization to ensure the appropriate matching of enamel oxygen values with drinking water values, samples without city-level information were not used in the water/enamel relationship analysis. City-level geographical information could not be retrieved for 21 of the analyzed samples due to either missing information or where individuals provided only the name of the country of residence. A single sample from the Fiji Islands was included in the study, albeit without city-level information, because of the country’s relatively small size and as the only sample from Oceania. Lastly, five samples came from individuals who had relocated multiple times during the span of tissue formation and could not be associated with any single city. A total of 75 enamel samples with reliable geographical associations remained for interpretation.

A summary of *δ*^18^O_c_ results for all analyzed samples is provided as Supplementary Table S1 online along with the corresponding mean *δ*^18^O_dw_ values taken from other published studies for the city of residence. Estimated precipitation oxygen values using the OIPC (*δ*^18^O_OIPC_) and Regionalized Cluster-based Water Isotope Prediction (*δ*^18^O_RCWIP_) are also included. The estimated *δ*^18^O_OIPC_ and *δ*^18^O_RCWIP_ values were not statistically significantly different as per the pairwise t-test [t(74) = – 0.747, df = 74, p = 0.457], and thus only the estimated values from the OIPC were used for subsequent analyses.

### Enamel and tap water oxygen results for MV

**Figure 3:**
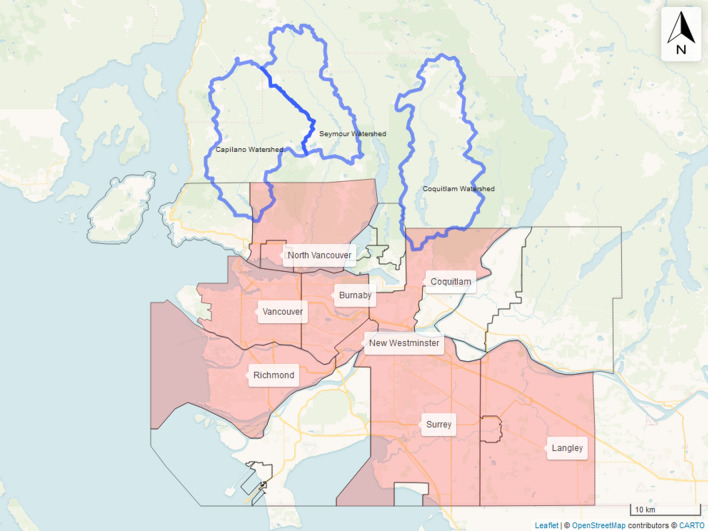
Map of MV with the shaded areas representing sampled municipalities. This map was generated using R version 4.0.3 (https://www.R-project.org/)^58,81^.

**Figure 4:**
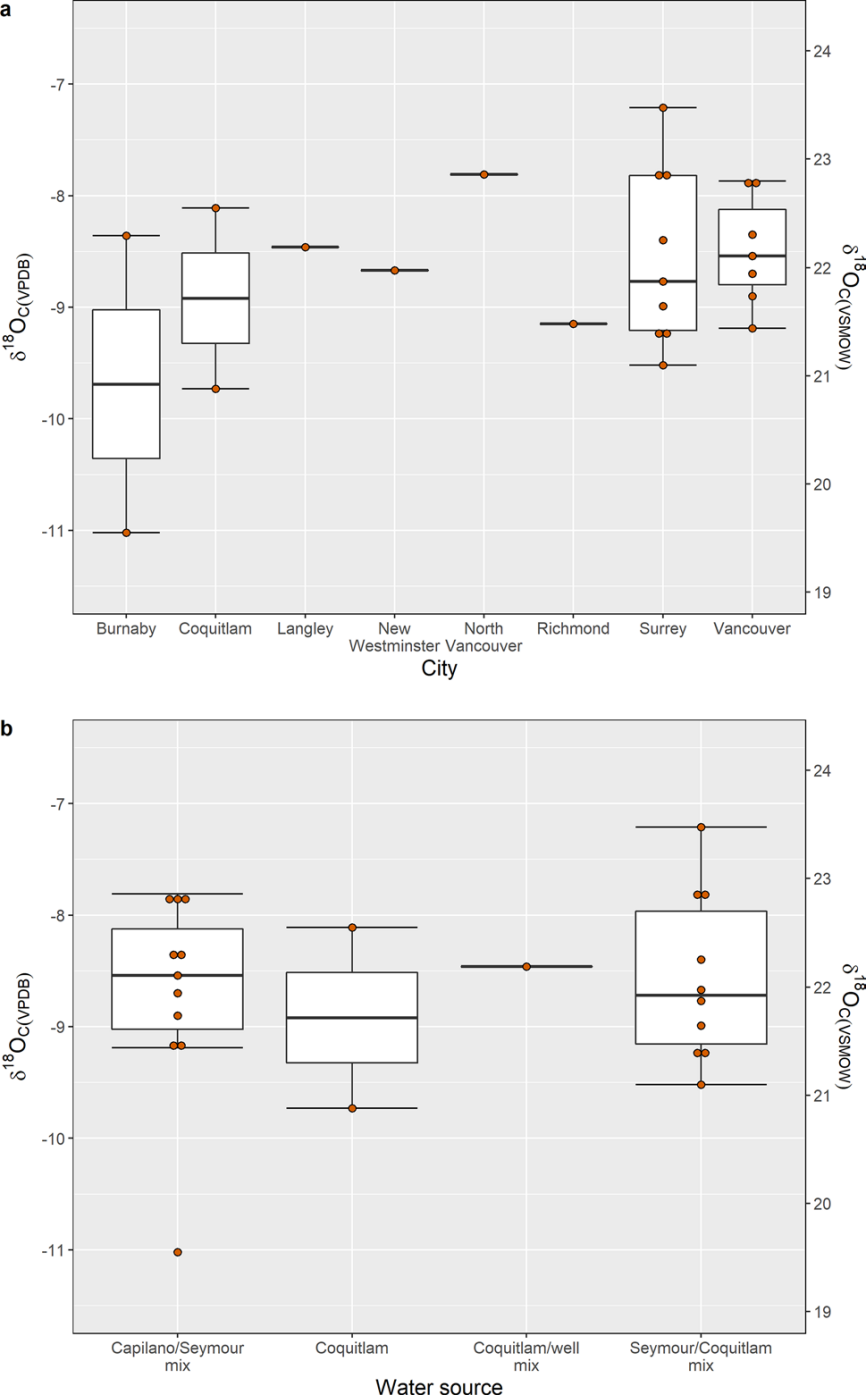
Boxplots of MV *δ*^18^O_C_ values as represented by (**a**) city and (**b**) tap water source. The boxes represent 25th and 75th percentiles, and whiskers represent the maximum and minimum observations. When outliers are present, the whiskers extend to 1.5 times the interquartile range. The middle line denotes the median or 50^th^ percentile. One-way ANOVA tests showed no statistically significant inter-city differences between mean *δ*^18^O_c_ values for MV [F(7, 16) = 0.717, p = 6.59], nor for mean MV *δ*^18^O_c(VPDB)_ values for data disaggregated by tap water source [F(4,19) = 1.013, p = 0.426].

24 individuals were of MV origin and covered eight municipalities—Burnaby, Coquitlam, Langley, Richmond, New Westminster, North Vancouver, Surrey, and Vancouver (Fig. 3). *δ*^18^O_c(VPDB)_ values ranged from − 11.0‰ (Burnaby) to − 7.2‰ (Surrey) with a mean value of − 8.7‰ ± 0.8 (Fig. 4a). One-way ANOVA test showed no statistically significant inter-city differences between mean *δ*^18^O_c(VPDB)_ values [F(7, 16) = 0.717, p = 6.59]. Further, no statistically significant differences were observed for mean *δ*^18^O_c(VPDB)_ values when data was disaggregated by tap water source [F(4,19) = 1.013, p = 0.426] (Fig. 4b), although an outlier was identified for the sample with *δ*^18^O_c(VPDB)_ value of − 11.0‰.

**Figure 5:**
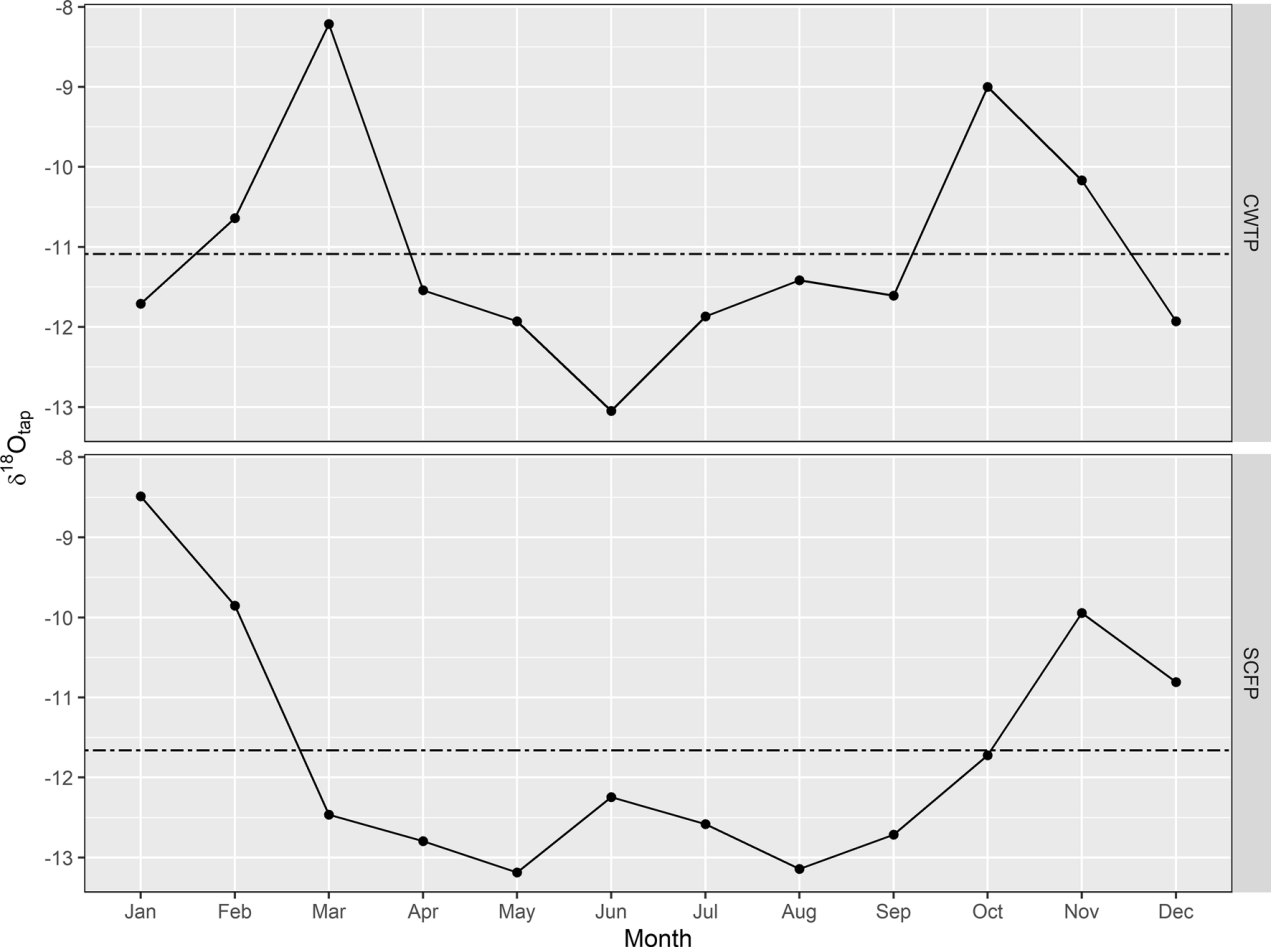
Monthly MV tap water *δ*^18^O data for samples taken between June 2017 to May 2018 at Coquitlam Water Treatment Plant (CWTP) and Seymour-Capilano Filtration Plant (SCFP). Mean annual MV tap water *δ*^18^O values were – 11.1‰ and – 11.7‰ at CWTP and SCFP, respectively.

Monthly tap water oxygen (*δ*^18^O_tap_) values ranged from – 13.0 to –8.2‰ with an annual mean value of – 11.1‰ (n = 22), and – 13.2‰ to –8.5‰ with an annual mean value of – 11.7‰ (n = 23) for MV tap waters treated at CWTP and SCFP, respectively (Fig. 5). Fractional contributions of each major MV tap water source to the overall municipal water supply were calculated for all municipalities represented by the enamel samples (Table 4).

**Table 4:** Fractional contributions of water sources to MV municipal tap water supply.

Mean annual * δ*^18^O_tap_	Capilano	Seymour	Coquitlam	Well	*δ*^18^O_(mean)_
	− 11.6	− 11.6	− 11.1	− 11.1	
Municipality	Fractional contributions (*f*)				
Burnaby	0.045	0.955	0	0	− 11.7
Coquitlam	0	0	1.00	0	− 11.1
Langley and Township of Langley	0	0	0.47	0.53	− 11.1
New Westminster	0	0.20	0.80	0	− 11.2
North Vancouver	0.50	0.50	0.00	0.00	− 11.7
Richmond	0.495	0.495	0.00	0.00	− 11.6
Surrey	0.00	0.41	0.59	0.00	− 11.3
Vancouver	0.62	0.38	0.00	0.00	− 11.7

The OLS regression for MV *δ*^18^O_c_ and *δ*^18^O_tap_ values yielded the result:

1 $$\delta^{{{18}}} {\text{O}}_{{{\text{c}}({\text{VSMOW}})}} = \, 0.{1}0 \, \left( { \pm 0.{75}} \right)\, \times \delta^{{{18}}} {\text{O}}_{{{\text{tap}}\left( {{\text{VSMOW}}} \right)}} + { 23}.0{8 }\left( { \pm {8}.{58}} \right); \, [{\text{df }} = { 22};\overline{R}^{2} = \, - 0.0{4}]$$

The negative $\overline{R }$^2^ value indicates that the data cannot be explained by the model.

### Enamel oxygen results for Canada

**Figure 6:**
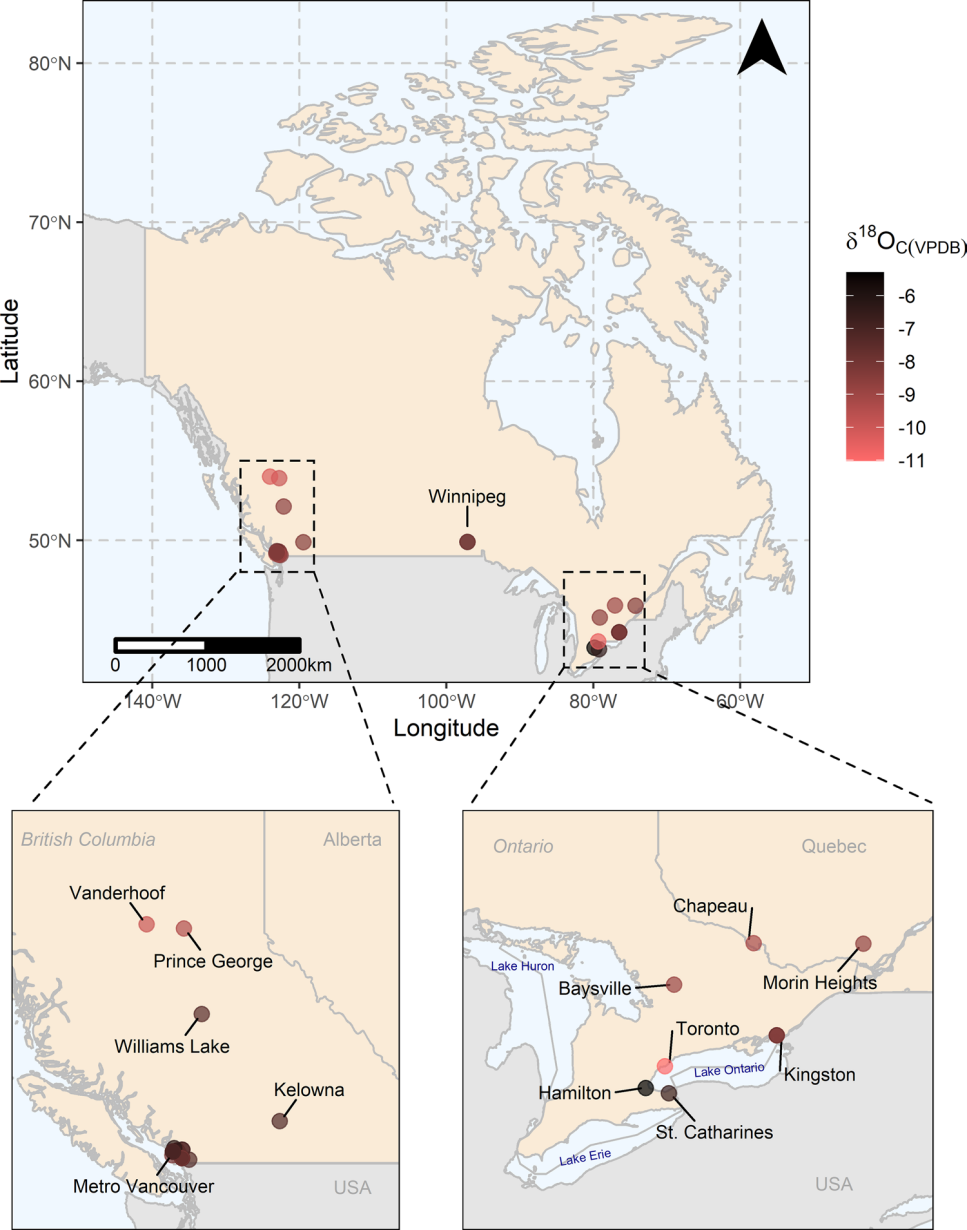
Location of residence during tissue formation with the corresponding *δ*^18^O_C(VPDB)_ values for individuals from across Canada. This map was generated using R version 4.0.3 (https://www.R-project.org/)^58^.

In addition to the 24 individuals from MV, four individuals were from BC cities outside of MV, two from Manitoba (MB), seven from Ontario (ON), and two from Quebec (QC) (Fig. 6). The mean *δ*^18^O_c(VPDB)_ value for BC was at − 8.7‰ ± 0.8 (n = 29), similar to the mean value for MV. The mean *δ*^18^O_c(VPDB)_ value for all Canadian samples was also similar at − 8.7‰ ± 1.1 (n = 41) with a range of − 11.0‰ (Burnaby, BC) to − 5.3‰ (Hamilton, ON). One-way ANOVA test showed no statistically significant differences between provinces [F(3,35) = 0.825, p = 0.489]. Cities supplied by Lake Ontario showed the largest range in *δ*^18^O_c(VPDB)_ values from – 10.6 to – 5.3‰ compared to other water sources from across Canada.

Source water information was retrieved for each represented city^52,62–67^. No source water information could be retrieved for Chapeau, QC, but the most probable water source was identified to be Ottawa River^68^. Modern *δ*^18^O_tap_ data were found for Hamilton, St. Catharines, Toronto^69^, and Winnipeg^70^ with given source information (see Supplementary Table S2 online). *δ*^18^O_tap_ data for Ottawa was taken for both Chapeau and Morin Heights as no *δ*^18^O_tap_ data were found for the two cities. Ottawa River supplies both Chapeau and Morin Heights, and which also is the water source for Ottawa. Only surface water values were found for the drinking water sources of Kelowna^71^, Prince George, and Vanderhoof^72^, and no *δ*^18^O_dw_ values were associated with Williams Lake and Baysville.

**Figure 7:**
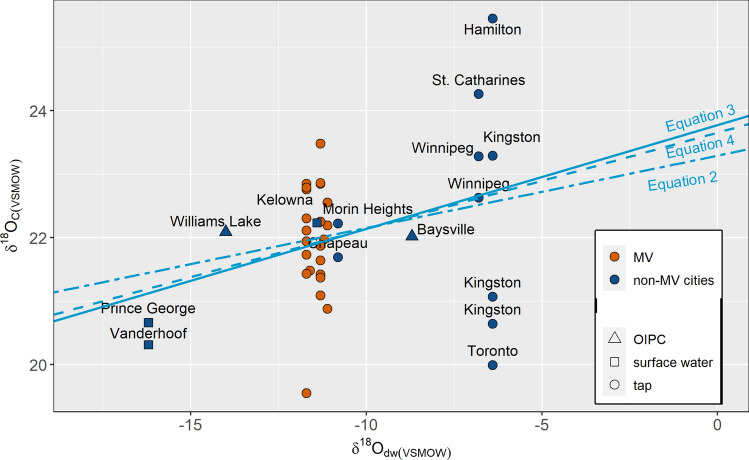
The relationships between *δ*^18^O_c(VSMOW)_ and *δ*^18^O_dw_ values are shown for individuals who had resided in Canada during tissue formation (n = 41). Colours indicate the MV or non-MV residence, and shapes indicate the type of drinking water sampled for its *δ*^18^O_dw_ value. The different line types show the regression line between *δ*^18^O_c_ and *δ*^18^O_tap_ values for Eq. (2): *δ*^18^O_c(VSMOW)_ = 0.11 (± 0.09) × *δ*^18^O_tap(VSMOW)_ + 23.29 (± 0.98); [df = 32; $\overline{R }$^2^ = 0.01], *δ*^18^O_dw_ values including both tap and surface water values for Eq. (3): *δ*^18^O_c(VSMOW)_ = 0.16 (± 0.08) × *δ*^18^O_dw_1(VSMOW)_ + 23.77 (± 0.82); [df = 35; $\overline{R }$^2^ = 0.09] and *δ*^18^O_dw_ values for all samples for Eq. (4): *δ*^18^O_c(VSMOW)_ = 0.15 (± 0.07) × *δ*^18^O_dw_2(VSMOW)_ + 23.65 (± 0.78); [df = 37; $\overline{R }$^2^ = 0.08].

*δ*^18^O_c(VSMOW)_ values were regressed against three sets of *δ*^18^O_dw_ values—(1) tap water values, (2) tap water and surface water or groundwater values (*δ*^18^O_dw_1_), and (3) a combined set of tap water, surface water or groundwater and OIPC values (*δ*^18^O_dw_2_) (Fig. 7) as not all samples could be directly paired with modern *δ*^18^O_tap_ values.

2 $$\delta^{{{18}}} {\text{O}}_{{{\text{c}}({\text{VSMOW}})}} = \, 0.{11 }\left( { \pm 0.0{9}} \right) \, \times \delta^{{{18}}} {\text{O}}_{{{\text{tap}}\left( {{\text{VSMOW}}} \right)}} + { 23}.{29 }\left( { \pm 0.{98}} \right); \, [{\text{df }} = { 32};\overline{R}^{2} = \, 0.0{1}]$$

3 $$\delta^{{{18}}} {\text{O}}_{{{\text{c}}({\text{VSMOW}})}} = \, 0.{16 }\left( { \pm 0.0{8}} \right)\, \times \delta^{{{18}}} {\text{O}}_{{{\text{dw}}\_{1}\left( {{\text{VSMOW}}} \right)}} + { 23}.{77 }\left( { \pm 0.{82}} \right); [{\text{df }} = { 35};\overline{R}^{2} = \, 0.0{9}]$$

4 $$\delta^{{{18}}} {\text{O}}_{{{\text{c}}({\text{VSMOW}})}} = \, 0.{15 }\left( { \pm 0.0{7}} \right)\, \times \delta^{{{18}}} {\text{O}}_{{{\text{dw}}\_{2}\left( {{\text{VSMOW}}} \right)}} + { 23}.{65 }\left( { \pm 0.{78}} \right); [{\text{df }} = { 37};\overline{R}^{2} = \, 0.0{8}]$$

The Analysis of Covariance (ANCOVA) test was used to compare the three sets of linear regressions to determine whether they significantly differed from each other. Results showed no statistically significant differences between the three equations, [F(2, 106) = 0.010, p = 0.990]. Equation (3) with the highest $\overline{R }$^2^ value proved to be the most reliable equation for Canada. Overall, although with a positive slope, the low $\overline{R }$^2^ values signify that the model cannot explain the relationship between the isotopic compositions of enamel oxygen and drinking water oxygen values for the Canadian samples. The dataset will need updating with increased sample collection.

### Enamel oxygen results for the global dataset

**Figure 8:**
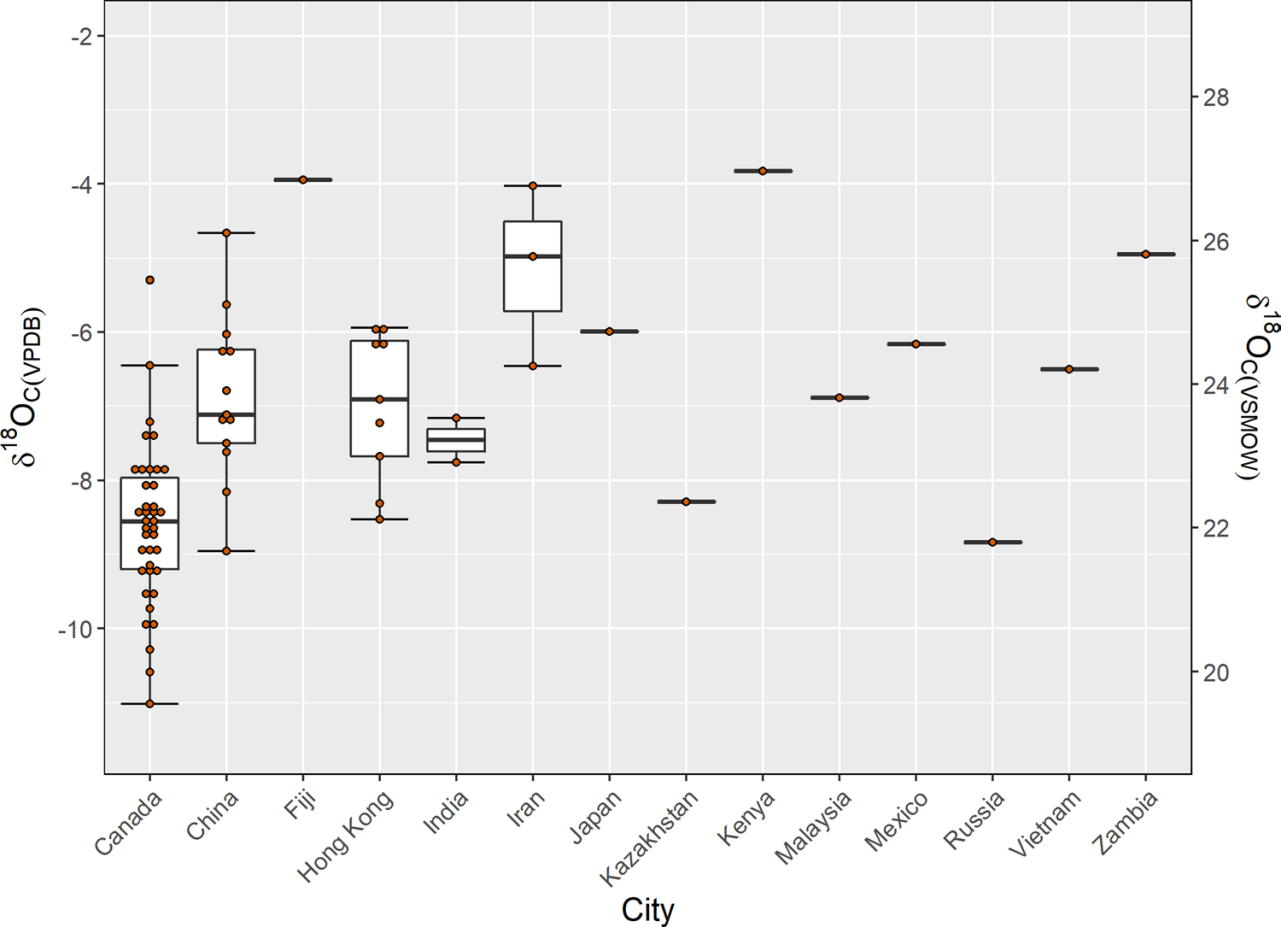
A boxplot of *δ*^18^O_c_ values for the globally aggregated data represented by country (n = 75). The boxes represent 25th and 75th percentiles, and whiskers represent the maximum and minimum observations. When outliers are present, the whiskers extend to 1.5 times the interquartile range. The middle line denotes the median or 50^th^ percentile.

**Figure 9:**
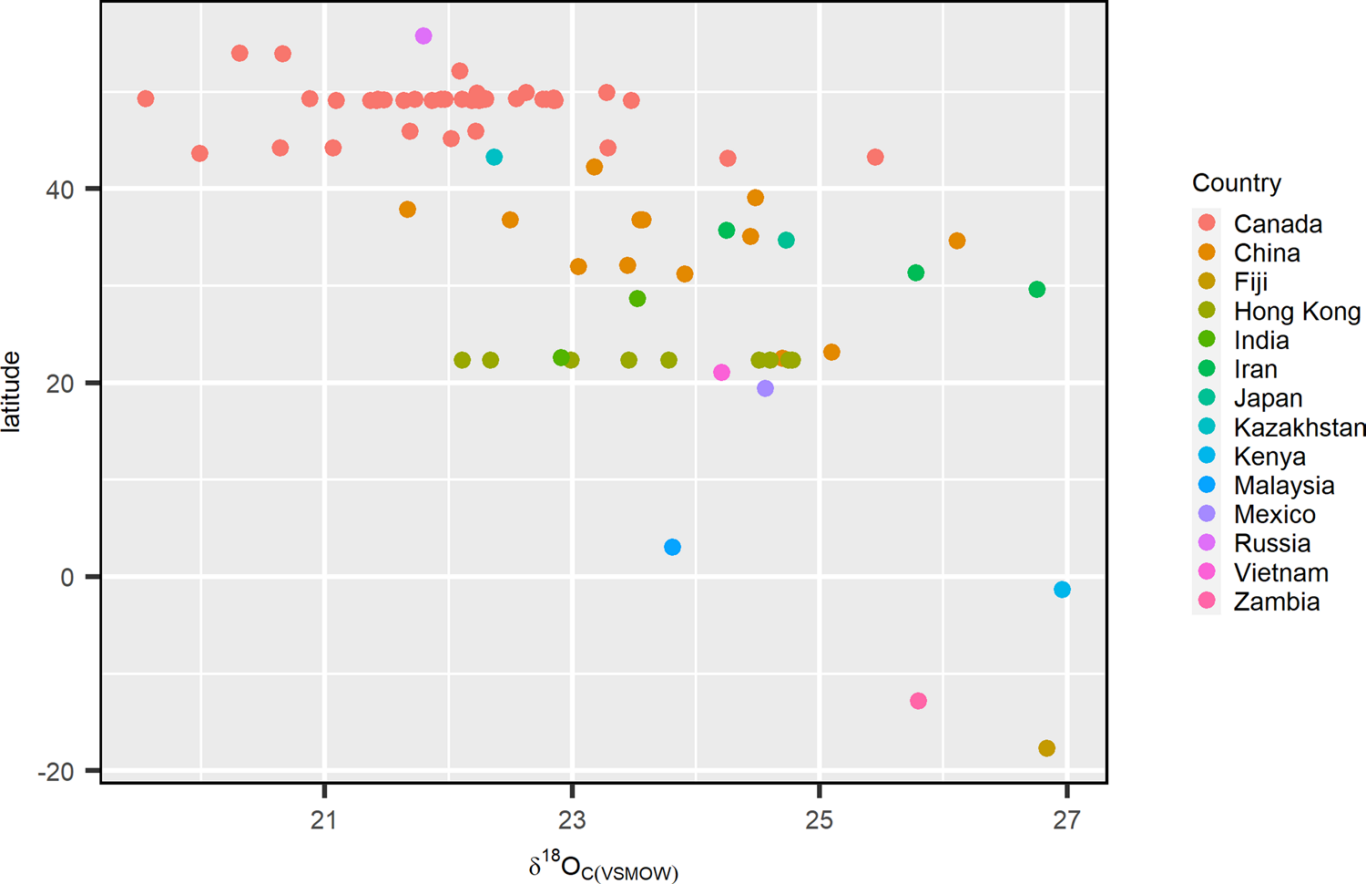
A plot of latitude vs *δ*^18^O_c(VSMOW)_ of all data (n = 75). A general latitudinal trend can be observed with *δ*^18^O_c_ values increasing decreasing latitude.

The remaining 36 samples included individuals from 13 additional countries encompassing the latitudinal areas from 55.75124 (Moscow, Russia) to – 17.71337 (Fiji Islands). The mean *δ*^18^O_c(VPDB_) values for all countries except for the single enamel formed in Russia were more negative in comparison to Canadian values (Fig. 8). There was a general latitudinal trend where *δ*^18^O_c_ values increased with decreasing latitude, although with a large range at higher latitudes (Fig. 9).

Tap water data were retrieved for China^37,46,73^, Hong Kong^46,73^, Iran, Kazakhstan, Kenya^73^, Malaysia^74^, Mexico, Russia, and Vietnam^73^ (see Supplementary Table S2 online). While tap water data was available for New Delhi, India^73,75^, only groundwater values could be retrieved for Kolkata, India^73^. No tap water value was found for Kitwe, Zambia; however, surface water data of Kafue River, which is the drinking water source for Kitwe^76^, was retrieved^77^. No such data were found for Fiji, Japan, and for Yiyuan, Zibo in China.

**Figure 10:**
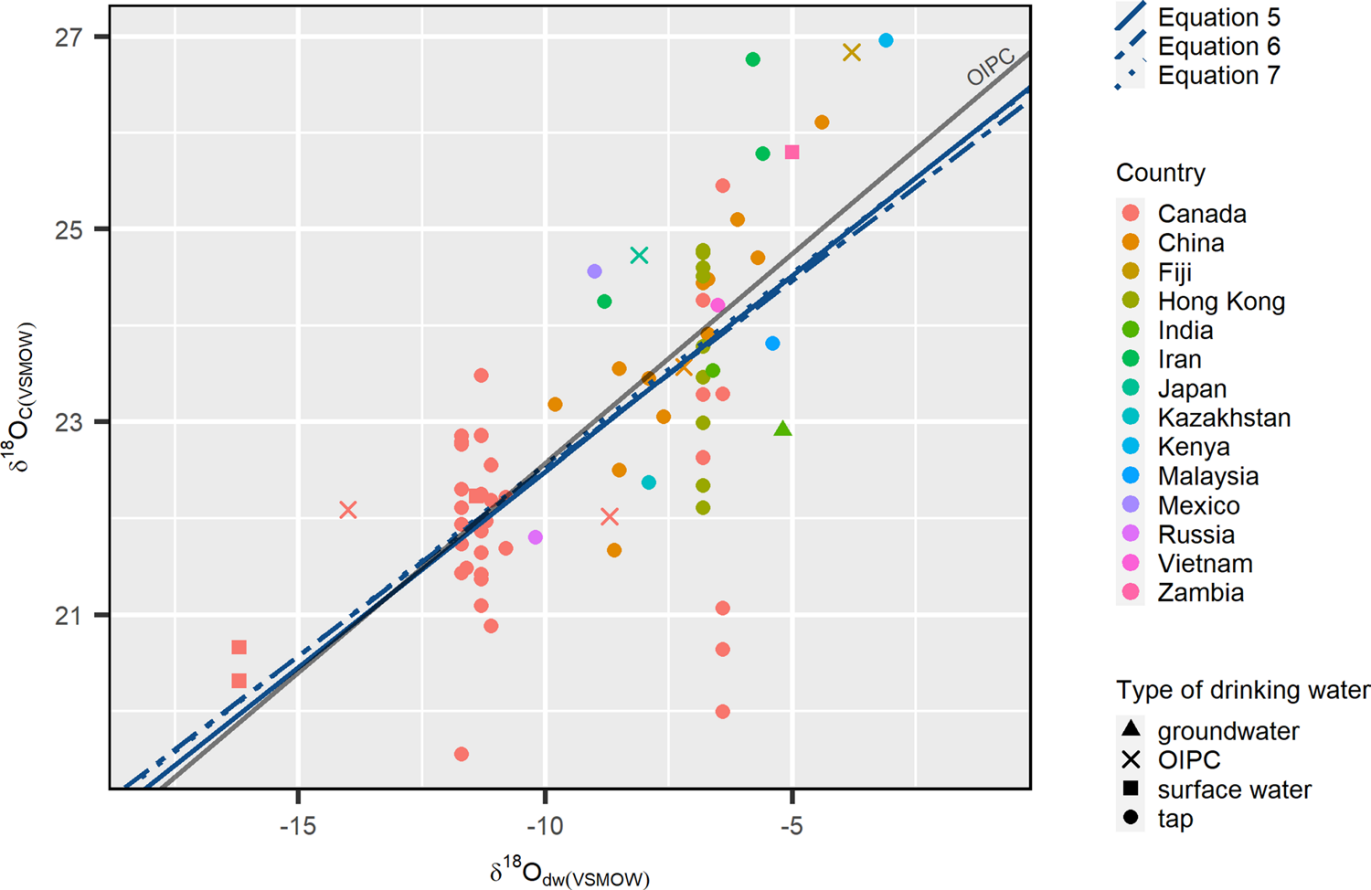
The relationships between *δ*^18^O_c(VSMOW)_ and *δ*^18^O_dw_ values are shown for all sampled individuals (n = 75). Colours indicate the country of residence, and shapes indicate the type of drinking water sampled for its *δ*^18^O_dw_ value. The different line types show the regression line between *δ*^18^O_c_ and *δ*^18^O_tap_ values for Eq. (5): *δ*^18^O_c(VSMOW)_ = 0.11 (± 0.09) x *δ*^18^O_tap(VSMOW)_ + 23.29 (± 0.98); [df = 32; $\overline{R }$^2^ = 0.01], *δ*^18^O_dw_ values including both tap and surface water values for Eq. (6): *δ*^18^O_c(VSMOW)_ = 0.16 (± 0.08) × *δ*^18^O_dw_1(VSMOW)_ + 23.77 (± 0.82); [df = 35; $\overline{R }$^2^ = 0.09] and *δ*^18^O_dw_ values for all samples for Eq. (7): *δ*^18^O_c(VSMOW)_ = 0.15 (± 0.07) x *δ*^18^O_dw_2(VSMOW)_ + 23.65 (± 0.78); [df = 37; $\overline{R }$^2^ = 0.08]. The solid grey line represents the regression line between *δ*^18^O_c_ and *δ*^18^O_OIPC_ values; Eq. (8): *δ*^18^O_c(VSMOW)_ = 0.43 (± 0.05) × *δ*^18^O_OIPC(VSMOW)_ + 26.92 (± 0.46); [df = 73,$\overline{R }$^2^ = 0.51].

The OLS regression for *δ*^18^O_c_ and *δ*^18^O_tap_ for the global dataset yielded the result (Fig. 10):

5 $$\delta^{{{18}}} {\text{O}}_{{{\text{c}}({\text{VSMOW}})}} = \, 0.{41 }\left( { \pm 0.0{6}} \right)\, \times \delta^{{{18}}} {\text{O}}_{{{\text{tap}}({\text{VSMOW}})}} + { 26}.{55 }( \pm 0.{55}); \, [{\text{df }} = { 63},\overline{R}^{2} = \, 0.{41}]$$

Similar to the Canadian dataset, *δ*^18^O_c(VSMOW)_ values were regressed against *δ*^18^O_dw_1(VSMOW)_, and *δ*^18^O_dw_2(VSMOW)_ values, as well as against *δ*^18^O_OIPC(VSMOW)_.

6 $$\delta^{{{18}}} {\text{O}}_{{{\text{c}}({\text{VSMOW}})}} = \, 0.{39 }\left( { \pm 0.0{5}} \right) \, \times \delta^{{{18}}} {\text{O}}_{{{\text{dw}}\_{1}({\text{VSMOW}})}} + { 26}.{41 }( \pm 0.{47}); \, [{\text{df }} = { 68},\overline{R}^{2} = \, 0.{45}]$$

7 $$\delta^{{{18}}} {\text{O}}_{{{\text{c}}({\text{VSMOW}})}} = 0.{4}0 \, \left( { \pm 0.0{5}} \right)\, \times \delta^{{{18}}} {\text{O}}_{{{\text{dw}}\_{2}({\text{VSMOW}})}} + { 26}.{53} ( \pm 0.{45}); \, [{\text{df }} = { 73},\overline{R}^{2} = \, 0.{47}]$$

8 $$\delta^{{{18}}} {\text{O}}_{{{\text{c}}({\text{VSMOW}})}} = 0.{43 }\left( { \pm 0.0{5}} \right)\, \times \delta^{{{18}}} {\text{O}}_{{{\text{OIPC}}({\text{VSMOW}})}} + { 26}.{92 }( \pm 0.{46}); \, [{\text{df }} = { 73},\overline{R}^{2} = \, 0.{51}]$$

The ANCOVA test showed no statistically significant differences between the four equations [F(3, 280) = 0.144, p = 0.933]. Of the four equations, Eq. (8) had the highest $\overline{R }$^2^ value and thus proved to be the most reliable equation for the global dataset.

### Predictability of existing models

**Figure 11:**
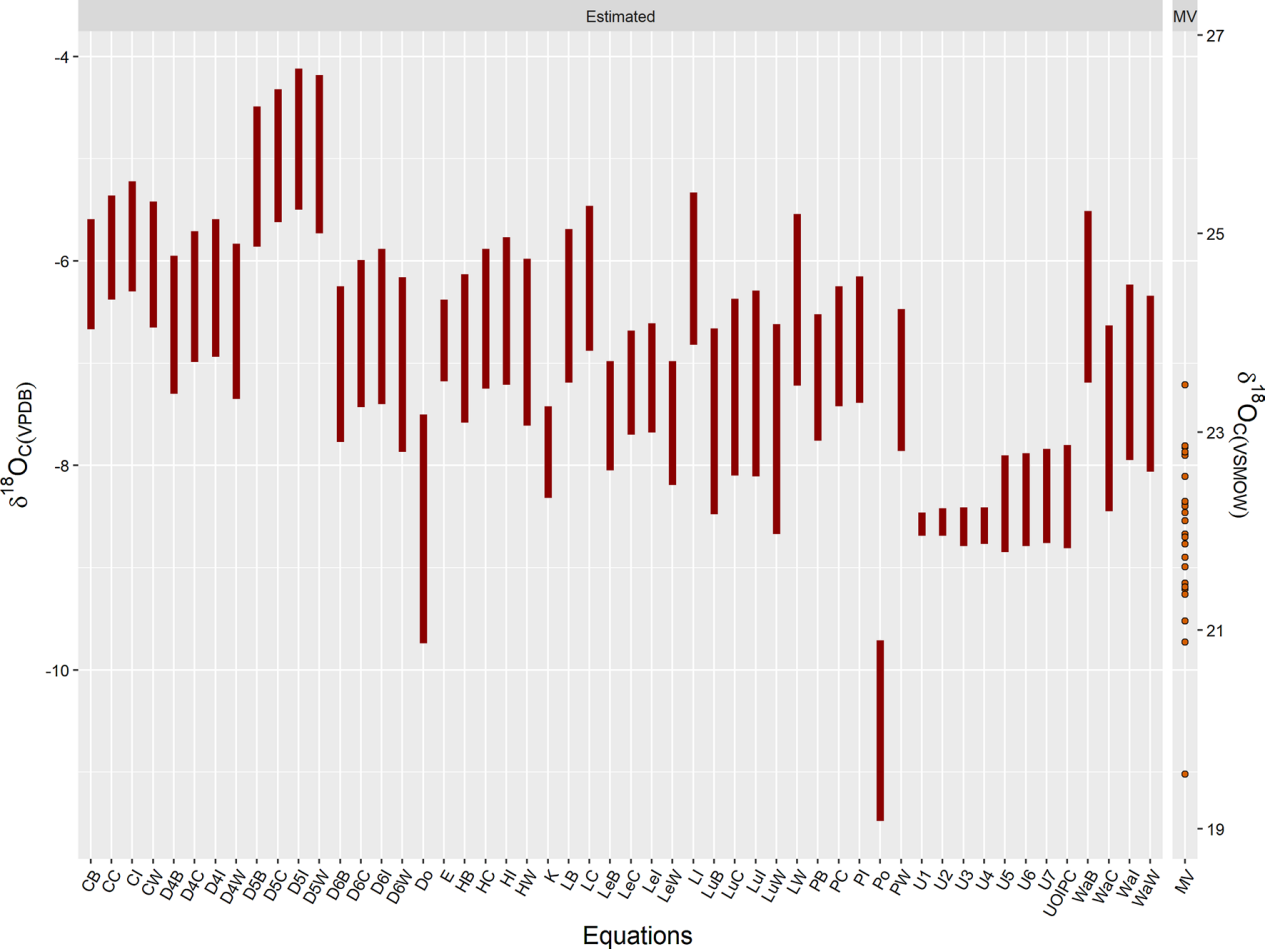
Range of *δ*^18^O_c_ values as predicted by an equation or a combination of equations from the actual Metro Vancouver (MV) *δ*^18^O_tap_ range (− 11.7 to − 9.3‰). Equations are shown by their coded names (Table 5) and include all existing predictive models as well as equations from the current study (U1, U2, U3, U4, U5, U6, U7, UOIPC). Predicted ranges were generally more positive than the actual range for MV. 15 of the 44 predicted ranges fell outside the actual MV range. The best performing predictive model was Dotsika^40^ (Do), which accurately identified 91.7% of the MV individuals residing in MV during tissue formation.

The ranges of MV *δ*^18^O_c_ values that were predicted from each water/enamel conversion model are shown in Fig. 11. Actual MV *δ*^18^O_tap_ values were used for the calculations. Water/enamel equations based on phosphate oxygen were paired with a phosphate/carbonate equation when conversions from phosphate oxygen to drinking water oxygen were necessary. All combinations of models are listed in Table 5 along with the abbreviated codes used in Fig. 11. The minimum MV tap water oxygen value of – 11.7‰ was taken from annualized data of SCWT, and the maximum value of – 9.3‰ was reported in Ueda and Bell^47^ from a groundwater source. The range of values estimated by Dotsika’s^17^ equation provided the most accurate estimate where 91.7% of MV individuals were correctly identified to have resided in MV during their childhood years. 15 combinations of models failed to identify any MV individuals accurately. The observed ranges of *δ*^18^O_c_ values for MV were generally more negative than predicted ranges from existing models except for ranges predicted with Posey’s^22^ equation (Fig. 11).

**Table 5:** Abbreviations for conversion models.

Model	Paired * δ*^18^O_p_/*δ*^18^O_c_ equation	CODE
Chenery et al.^18^	Bryant et al.^30^	CB
Chenery et al.^18^	Chenery et al.^24^	CC
Chenery et al.^18^	Iacumin et al.^31^	CI
Chenery et al.^18^	Wright et al.^32^	CW
Daux et al.^16^ (Eq. 4)	Bryant et al.^30^	D4B
Daux et al.^16^ (Eq. 4)	Chenery et al.^24^	D4C
Daux et al.^16^ (Eq. 4)	Iacumin et al.^31^	D4I
Daux et al.^16^ (Eq. 4)	Wright et al.^32^	D4W
Daux et al.^16^ (Eq. 5)	Bryant et al.^30^	D5B
Daux et al.^16^ (Eq. 5)	Chenery et al.^24^	D5C
Daux et al.^16^ (Eq. 5)	Iacumin et al.^31^	D5I
Daux et al.^16^ (Eq. 5)	Wright et al.^32^	D5W
Daux et al.^16^ (Eq. 6)	Bryant et al.^30^	D6B
Daux et al.^16^ (Eq. 6)	Chenery et al.^24^	D6C
Daux et al.^16^ (Eq. 6)	Iacumin et al.^31^	D6I
Daux et al.^16^ (Eq. 6)	Wright et al.^32^	D6W
Dotsika ^17^	NA	Do
Ehleringer et al.^14^	NA	E
Herrmann et al.^19^	Bryant et al.^30^	HB
Herrmann et al.^19^	Chenery et al.^24^	HC
Herrmann et al.^19^	Iacumin et al.^31^	HI
Herrmann et al.^19^	Wright et al.^32^	HW
Keller et al.^20^	NA	K
Levinson et al.^8^	Bryant et al.^30^	LeB
Levinson et al.^8^	Chenery et al.^24^	LeC
Levinson et al.^8^	Iacumin et al.^31^	LeI
Levinson et al.^8^	Wright et al.^32^	LeW
Longinelli ^6^	Bryant et al.^30^	LB
Longinelli ^6^	Chenery et al.^24^	LC
Longinelli ^6^	Iacumin et al.^31^	LI
Longinelli ^6^	Wright et al.^32^	LW
Luz et al.^7^	Bryant et al.^30^	LuB
Luz et al.^7^	Chenery et al.^24^	LuC
Luz et al.^7^	Iacumin et al.^31^	LuI
Luz et al.^7^	Wright et al.^32^	LuW
Pollard et al.^21^	Bryant et al.^30^	PB
Pollard et al.^21^	Chenery et al.^24^	PC
Pollard et al.^21^	Iacumin et al.^31^	PI
Pollard et al.^21^	Wright et al.^32^	PW
Posey^22^	NA	Po
Ueda_1	NA	U1
Ueda_2	NA	U2
Ueda_3	NA	U3
Ueda_4	NA	U4
Ueda_5	NA	U5
Ueda_6	NA	U6
Ueda_7	NA	U7
Ueda_OIPC	NA	UOIPC
Warner et al.^23^	Bryant et al.^30^	WaB
Warner et al.^23^	Chenery et al.^24^	WaC
Warner et al.^23^	Iacumin et al.^31^	WaI
Warner et al.^23^	Wright et al.^32^	WaW

**Figure 12:**
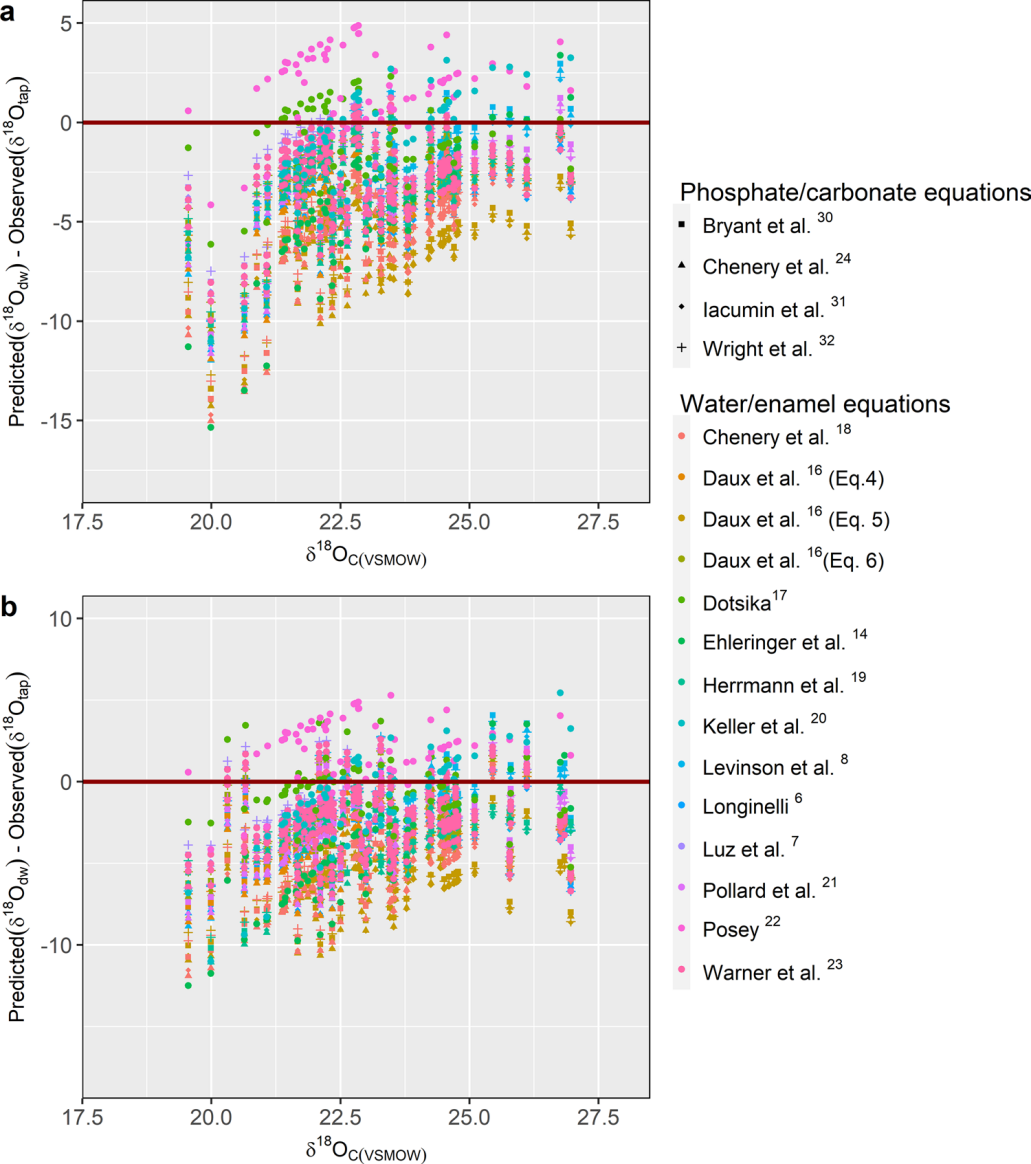
This graph provides a visual representation of the offsets between predicted and observed drinking water values calculated using existing water/enamel conversion equations. Differences between predicted drinking water oxygen (*δ*^18^O_dw_) values and observed drinking water oxygen values as measured by (**a**) tap water (*δ*^18^O_tap_) and (**b**) OIPC (*δ*^18^O_OIPC_) for all samples from the current study (n = 75). The best predictors fall closest to 0 (solid line), which occurs when predicted drinking water values equal observed drinking water values. *δ*^18^O_dw_ values were predicted from actual enamel carbonate oxygen values using existing water/enamel conversion equations indicated by the various colours. Phosphate/carbonate conversion equations were applied to water/enamel equations when direct water/enamel conversions were not possible. Shapes denote applied phosphate/conversion equations.

**Figure 13:**
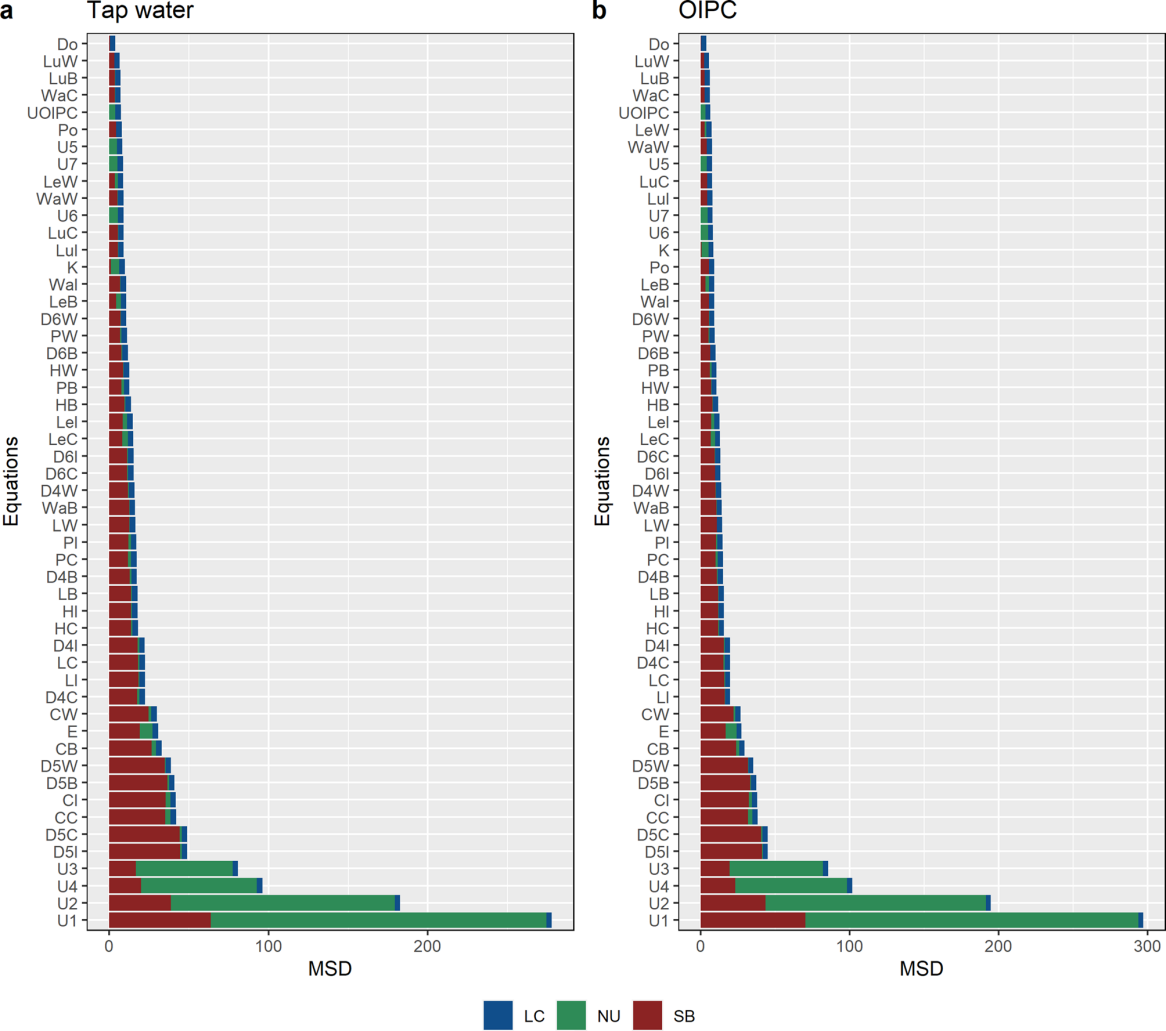
A direct comparison of the mean standard deviation (MSD) between predicted and observed *δ*^18^O_dw_ values for (**a**) tap water and (**b**) OIPC. *δ*^18^O_dw_ values were predicted from *δ*^18^O_c_ values of all samples (n = 75) from the current study with existing predictive models or a combination of models. MSD components are lack of correlation (LC), non-unity slope (NU) and squared bias (SB). Low MSD indicates greater similarity between modelled and measured values.

The offset between predicted and actual drinking water values ranged from – 15.3 to + 5.5‰ for tap waters (Fig. 12a) and – 12.5 to + 6.5‰ for OIPC (Fig. 12b). The use of the equation given by Dotsika^17^ produced the least MSD between predicted and observed drinking water values for both tap water measurements (MSD = 4.00; NU = 0.00; LC = 3.45; SB = 0.55) (Fig. 13a) and OIPC measurements (MSD = 3.72; NU = 0.07; LC = 3.42; SB = 0.23) (Fig. 13b; see Supplementary Table S4 online). Equation (1) gave the highest MSD for both tap water (MSD = 278.21; NU = 210.61; LC = 3.45; SB = 64.15) and OIPC measurements (MSD = 297.08; NU = 223.42; LC = 3.41; SB = 70.24).

## Discussion

The isotopic compositions of enamel samples collected from across Metro Vancouver scattered across an isotopic range of − 11.0 to − 7.2‰. This was similar to the predicted range of values calculated by Dotsika’s^17^ water/enamel conversion equation (Fig. 11). No statistically significant differences in mean *δ*^18^O_c_ values were observed across the Metro Vancouver municipalities indicating that geolocating humans based solely on *δ*^18^O_c_ values is difficult at the city-level for MV. The majority of MV enamel samples collected in this study covered areas supplied by the major surface water watersheds with similar annualized oxygen values and which source 95% of MV tap water. The isotopic compositions of oxygen in water from the major watersheds are lower in value than groundwater values. Thus individuals living in areas supplied by surface water sources should show lower enamel values compared to individuals from areas supplied by groundwater sources. The current dataset included a single individual who identified Langley as their place of residence during the time of tissue formation. Parts of the Township of Langley are supplied by groundwater sources but also include areas supplied by the surface water watersheds. The enamel oxygen value for the individual from Langley at − 8.5‰ was similar to the enamel oxygen values of individuals from areas supplied by surface water sources. With the lack of samples covering areas that may show the lowest enamel oxygen values for MV, it is unclear whether the current dataset has captured the entire range of MV enamel values. However, importantly, the dataset represents much of MV *δ*^18^O_c_ values and most definitely the lower limit of the range. Notably, understanding this lower limit signifies the possibility of oxygen isotope analysis to be used as an exclusionary tool to identify non-MV residents whose enamel oxygen isotope compositions lie below the most negative MV value of − 11.0‰.

Sampling across MV enabled the collection of a globally representative dataset due to the regional district’s high fluidity in both national and international migration^61^. The global representation of samples collected from one single regional district also highlights the highly mobility of humans and that we need to be cautious of presuming the location of tissue sampling to be the location of tissue formation. Notably, *δ*^18^O_c_ values in known modern samples from the entire MV region showed a distinct separation from other BC cities (Prince George and Vanderhoof) located at higher latitudes and signifies the possibility of utilizing oxygen isotope analysis to distinguish MV from northern BC cities. However, overlaps in *δ*^18^O_c_ values were observed with other Canadian cities regardless of the latitudinal differences (Fig. 7). This is contrary to the general latitudinal trend observed at the global level. Mean Canadian *δ*^18^O_c_ values were slightly more negative than *δ*^18^O_c_ values from individuals residing in other countries during the time of enamel formation, although with increasing overlap for the more positive *δ*^18^O_c_ values. This again underscores the potential for oxygen isotope analysis to be used as an exclusionary method for identifying individuals with a non-Canadian origin where *δ*^18^O_c_ values fall outside the Canadian range.

Overall, the equations derived from this study showed a smaller fractional contribution of drinking water oxygen to enamel oxygen compared to existing water/enamel models. The equations from the global dataset (Eqs. 5–8) were more comparable to other predictive models than those derived from higher geographical resolutions (Eqs. 1–4). Equation (8) derived from the global dataset with the highest $\overline{R }$^2^ was most comparable to Levinson et al.’s^8^ equation. The model performed better as the geographical resolution decreased from city to country to a global dataset. The reason for such a decrease in measured correlation between enamel and drinking water oxygen at a higher geographical resolution may mainly be due to the offsets observed between the isotopic compositions of oxygen in tap water and precipitation water for larger cities like Metro Vancouver relying on multiple water sources. Globally constructed predictive models should then be applied with caution at higher geographical resolutions as individuals can significantly deviate from the predicted regression line. A high-resolution study of tap waters may be necessary to understand the isotopic spread of water sources that supply the targeted regional area if supplied by water sources with isotopically distinct oxygen values. Like MV, enamel samples formed in Hong Kong showed a wide range of *δ*^18^O_c_ values, which only became evident with the concentrated number of individuals within our sample set that had resided in Hong Kong during the time of tissue formation. Hong Kong relies on both precipitation water and transported surface water from multiple sources^78^ with possibly distinct oxygen isotope compositions.

The predictability of existing water/enamel conversion equations was assessed through mean standard deviation calculations between predicted and observed drinking water values. Dotsika’s^17^ equation proved to best predict *δ*^18^O_dw_ from measured *δ*^18^O_c_ values with the lowest mean standard deviation of 4.00 for tap water values and 3.72 for OIPC water values. This could be attributed to the careful sampling of enamel with known geographical data paired with known drinking water data. The equations with the highest mean standard deviation measures were of those from this current study (Eqs. 1–4) with large NU components. NU is a direct measure of the slope where NU > 0 when the slope ≠ 1^60^. The large NU measures for Eqs. 1–4 can be explained by the low $\overline{R }$^2^ values, indicating the difficulty in determining the correlation between oxygen in drinking water and enamel at a higher geographical resolution.

Daux et al.’s^16^ equation (5) paired with Iacumin et al.’s^31^ equation (D5I) showed the largest MSD amongst the existing predictive models with a high SB score (Fig. 13). SB > 0 when intercept ≠ 0 and thus reflects the vertical shifting of a function and in turn could be a reflection of inter-laboratory differences in measured enamel oxygen values. Inter-laboratory differences between phosphate oxygen values, as discussed by Chenery et al.^18^ are generally related to bio-phosphate preparation with either BiPO_4_ or Ag_3_PO_4_ prior to oxygen extraction. Chenery et al. concluded that a correction of 1.4 to Levinson et al.’s^8^ equation is required to best predict local *δ*^18^O_dw_ values measured through the UK drinking water isoscapes. However, MSD calculations for this current study showed contrary results where Levinson et al.’s original equation performed better for known modern samples than that of Chenery et al.’s corrected Levinson’s equation. Overall, SB measures were generally lower for equations derived from carbonate oxygen analysis than those derived from phosphate oxygen prepared with BiPO_4_. Equations derived from phosphate oxygen prepared with Ag_3_PO_4_ had larger SB measures, as was the case for Daux et al.’s equations.

Differences in the predicted and observed *δ*^18^O_dw_ values were as high as 15‰, where differences increased with decreasing *δ*^18^O_c_ values (Fig. 12). Whereas the differences between predicted and observed *δ*^18^O_dw_ values generally fell within ± 5‰ for *δ*^18^O_c(VSMOW)_ values of 25 to 28‰, greater differences were observed for lower *δ*^18^O_c(VSMOW)_ values, with the highest difference observed for a sample with *δ*^18^O_c(VSMOW)_ value of 20‰. Inter-laboratory differences between *δ*^18^O_c_ values measured at three separate laboratories using different methodologies and instruments were < 1‰ for carbonate in tooth enamel^79^. Chesson et al.^33^ further tested for interlaboratory differences of enamel oxygen from the same set of samples analyzed a few years apart. The differences were mainly attributed to sample preparation and concluded that isotopic differences > 1.6‰ should be considered meaningful. The potential bias associated with inter-laboratory differences in sample preparation, methodology, and the choice of instruments, will further be compounded in the calculations of *δ*^18^O_dw_ values using water/enamel conversion equations. The compounding factor is dependent on the slope of the applied water/enamel equation. For example, Ehleringer’s^14^ equation with a slope of 1.12 would translate to a maximum of 1.8‰ difference in *δ*^18^O_dw_ values that may be associated with inter-laboratory differences. Ehleringer’s equation showed high offsets between observed *δ*^18^O_dw_ values and predicted *δ*^18^O_dw_ values for individuals with relatively more negative *δ*^18^O_c_ values. Such large differences beyond what can be explained by the possible inter-laboratory differences can be concluded to be important.

Differences in slope and intercept of existing equations may be attributed to various methodological approaches, such as in the types of sampled tissues in which the equations were based upon, the period of tissue formation, the spatial resolution of collection sites^6–8,14,16,18,21^ (Table 1). For example, one of the early models given by Longinelli^6^ was based on measurements of human bone bioapatite rather than on human enamel. Some studies lacked information on the specific types of sampled tooth^7,8^ while others studied archaeological or forensic samples with inferred geographical information where drinking water sources may be unknown. The oxygen isotope compositions of enamel can also be measured through the analysis of carbonate or phosphate oxygen. Thus, inter-laboratory differences cannot fully explain the observed differences between estimated drinking water values.

Large offsets in predicted values can lead to significant errors when inferring human mobility for identification purposes. The results from this study support the recommendation to standardize the methodology for the development of water/enamel equations, as stated in other papers^33,79^. There is undeniably a much-needed refinement of current predictive models, especially for utilizing conversion equations to develop human enamel oxygen isoscapes. The reliability of predictive models is based on the degree to which human tissues can be assigned to a geographical locale and the understanding of the true source of drinking water in which the individual had consumed during tissue formation. Models should thus be based on the oxygen analysis of modern human enamels with known geographical information for pairing with appropriate drinking water data. The preferred study is of known modern samples where geographical information can be obtained directly from the living donor over unknown or archaeological samples where inference of these types of information is required. Drinking water data should also be thoroughly examined, where tap water with known source information can best provide the most accurate drinking water data. Perhaps, an alternative approach to using a model-based approach could be a sample-based approach. Enamel oxygen values can be determined for specified geographical areas, and directly recorded onto a geographical map. The geographical assignment of individuals can then be determined through the use of classification trees or discriminant function models^80^. This approach requires all target regions to be defined a priori as categorical response variables and may require an extensive collection of samples with known sources. However, if the purpose of analyzing stable oxygen isotopes is to determine whether the individual is of interest to the local jurisdiction or not, then the categorical response variables can be determined in such a way that allows for the classification of local or non-local origin. A strategic approach to understanding the isotopic spread of enamel oxygen for that geographical region will then be necessary. A suggested approach would be to (1) gain an understanding of the drinking water source and distribution system for that region, and to (2) strategically obtain reference enamel samples from local areas that receive water from sources with oxygen values that lie on the most distal ends of the isotopic range for that region.

## Conclusion

This study aimed to assess the relationships between known biogeographical enamel dataset to a related known city-wide drinking water dataset. The results indicated that the water/enamel model cannot explain the relationship between *δ*^18^O_c_ and *δ*^18^O_tap_ values for Metro Vancouver at the city-level and also for Canada at the country-level. Mean standard deviation measurements were carried out to assess the predictability of existing conversion models. The equation provided by Dotsika^17^ proved to be the best model for this current set of enamel data. However, large differences between actual and predicted *δ*^18^O_dw_ values were noted from the use of existing conversion equations. Thus, this study supports the development of sample-based, or rather, *tissue-specific δ*^18^O_c_ geographical reference maps. Standardization of methodology for isotopic analysis is also vital for the collaborative collection of globally represented data. The development of international enamel oxygen isotope databases would ultimately render conversion equations unnecessary and eliminate any associated errors. A meaningful approach would be to define boundaries confined by the tap water distribution systems rather than by way of political borders, which can be made possible with the detailed understanding of tap water supply and distribution systems for any given geographical area. The development of a reliable database would greatly benefit the forensic community by providing accurate guidance for unidentified individuals’ possible region-of-origin determination. Furthermore, understanding the lower boundary of *δ*^18^O_c_ values for Metro Vancouver (− 11.0‰) now allows for oxygen isotope analysis to be used as an exclusionary tool where individuals with *δ*^18^O_c_ values falling below the threshold value can be identified as non-local. Such a tool is vital for assisting the forensic community in missing persons investigations.

## Supplementary Information

Supplementary Table S1.

Supplementary Table S2.

Supplementary Table S3.

Supplementary Table S4.

## Data Availability

All data analysed during the current study are available from the corresponding authors on reasonable request.
